# Real-world effectiveness of onabotulinumtoxinA treatment for the prevention of headaches in adults with chronic migraine in Australia: a retrospective study

**DOI:** 10.1186/s10194-019-1030-z

**Published:** 2019-07-15

**Authors:** Catherine Stark, Richard Stark, Nicole Limberg, Julian Rodrigues, Dennis Cordato, Raymond Schwartz, Robert Jukic

**Affiliations:** 1 Austin Health Heidelberg, 145 Studley Rd, Heidelberg, Victoria 3084 Australia; 2 Alfred Hospital and Monash University, 99 Commercial Rd, Melbourne, Victoria 3004 Australia; 3 Migraine Specialist, 33 North St.,, Spring Hill, QLD 4000 Australia; 4 Hollywood Private Hospital, 45/85 Monash Ave, Nedlands, WA 6009 Australia; 5 Southern Neurology and Healius Healthcare, 19 Kensington St.,, Kogarah, NSW 2217 Australia; 6 Allergan Australia Pty Ltd, 810 Pacific Hwy.,, Gordon, NSW 2072 Australia

**Keywords:** Migraine disorders, Headache, OnabotulinumtoxinA, Australia, Retrospective studies, Codeine, Chronic migraine

## Abstract

**Background:**

OnabotulinumtoxinA (BOTOX®, Allergan plc, Dublin, Ireland) is approved for the preventive treatment of headaches in adult patients with chronic migraine (CM) in Australia by the country’s reimbursement mechanism for medicines, the Pharmaceutical Benefits Scheme (PBS). To our knowledge, this study represents the first focused report evaluating real-world evidence of onabotulinumtoxinA treatment via the PBS in Australian clinics.

**Methods:**

This study reviewed the medical records of adults with inadequately controlled CM from 7 private neurology practices in Australia who, beginning in March 2014, received PBS-subsidized onabotulinumtoxinA per product labelling for the first time. The primary effectiveness measure was the percentage of patients achieving a response defined by 50% or greater reduction in headache days from baseline after 2 treatment cycles. Additional data were recorded in the case report form when available and included demographics, clinical characteristics, headache severity and frequency, Headache Impact Test (HIT-6) score, medication use, and days missed of work or study at baseline, after 2 treatment cycles, and at last follow-up. Differences in mean changes from baseline were evaluated with a 1-tailed *t*-test or Pearson’s chi-squared test (*p* < 0.05).

**Results:**

The study population included 211 patients with a mean (SD) of 25.2 (5.3) monthly headache days at baseline. In the primary outcome analysis, 74% of patients achieved a response, with a mean (SD) of 10.6 (7.9) headache days after 2 treatment cycles (*p* < 0.001). Secondary effectiveness outcomes included mean (SD) reductions in HIT-6 score of − 11.7 (9.8) and − 11.8 (12.2) after 2 treatment cycles (*p* < 0.001) and final follow-up (*p* < 0.001), respectively, and mean (SD) decreases in days per month of acute pain medication use of − 11.5 (7.6) after 2 treatment cycles (*p* < 0.001) and − 12.7 (8.1) at final follow-up (*p* < 0.001).

**Conclusion:**

This study provides additional clinical evidence for the consistent effectiveness of onabotulinumtoxinA for the treatment of CM in Australia. This effectiveness was made evident by reductions in migraine days, severe headache days, and HIT-6 scores from baseline.

## Background

Chronic migraine (CM) is a neurological disease that impacts approximately 1.4% to 2.2% of the global population [1]. CM is differentiated from episodic migraine by a more debilitating disease profile that includes a greater frequency of headache and migraine days and greater prevalence of anxiety and depression [2–4]. People with CM experience frequent disabling migraine attacks, preventing them from participating in daily activities and significantly impacting their quality of life [5]. CM is associated with a substantial societal and familial burden, with increased direct and indirect costs that can lead to a sizable economic burden for patients and healthcare systems [5–7].

Management of CM involves multiple factors including lifestyle modifications, trigger management, and effective utilization of acute and preventive medications [8]. Currently available oral migraine preventive drugs are associated with relatively low rates of persistence and adherence in people with migraine, limiting the effectiveness of treatment [9, 10]. OnabotulinumtoxinA (BOTOX®, Allergan plc, Dublin, Ireland) is approved in Australia for the preventive treatment of headaches in adults with CM and has been listed on Australia’s Pharmaceutical Benefits Scheme (PBS), the national medication reimbursement program, since March 2014. Prerequisites for receiving PBS-subsidized onabotulinumtoxinA treatment include a monthly headache frequency consistent with CM as defined by the International Classification of Headache Disorders, 3rd edition (ICHD-3) and failed viability of at least 3 prescribed preventive medications. Continued PBS reimbursement eligibility depends on achieving and maintaining a 50% or greater reduction from baseline in the number of headache days per month after two 12-week treatment cycles. This positive treatment response at 24 weeks aligns with results from the pivotal phase 3 Research Evaluating Migraine Prophylaxis Therapy (PREEMPT) randomized trials, which found onabotulinumtoxinA injections to be well tolerated and significantly more effective than placebo for reducing headache days at week 24 [11, 12]. These findings were further supported in the Chronic Migraine OnabotulinumtoxinA Prolonged Efficacy Open-Label (COMPEL) study, which evaluated longer-term use of onabotulinumtoxinA (108 weeks/9 cycles) in Australia, South Korea, and the United States [13].

Studies of CM treatments in clinical practice can help detect potential differences between the efficacy observed in randomized clinical trials and real-world effectiveness, which takes into account both efficacy and tolerability in the general patient population, including those not represented in controlled trials due to inclusion/exclusion criteria. This type of study would be of value in Australia because, to our knowledge, it would be the first study focused on the real-world effectiveness of PBS-subsidized onabotulinumtoxinA treatment in Australian clinics. Toward that goal, this multicentre retrospective chart review of medical records in Australia was undertaken to assess real-world experience with onabotulinumtoxinA for preventive treatment of CM in adults treated through the PBS.

## Methods

This study was conducted in accordance with the International Council for Harmonisation Guidelines on Good Clinical Practice and in accordance with the National Statement on Ethical Conduct in Human Research 2007 (updated May 2015). Investigators were required to obtain approval from a properly constituted Human Research Ethics Committee at their institutions prior to initiating the study.

### Study design

This was a multicentre, retrospective chart review of adults with CM in Australian clinical practice treated with onabotulinumtoxinA for headache prophylaxis to assess real-world treatment outcomes. Review and analysis of medical records from 7 private practices in the Australian Capital Territory and in the states of Victoria, Queensland, New South Wales, and Western Australia were conducted between April 2016 and January 2017. At least 2 treatment cycles were required to enable data extraction for a period spanning approximately 28 weeks.

### Patients

Patients in this study necessarily were eligible for PBS-subsidized onabotulinumtoxinA treatment for prevention of migraine, which means that they were aged 18 years or older, were under the care of a neurologist, and had experienced an average of 15 or more headache days per month, with at least 8 migraine days, over a period of 6 months or longer. The ICHD-3 definition of CM specifies the same monthly headache frequency, albeit with a lower temporal threshold – headache occurring on 15 or more days per month for more than 3 months [14]. PBS criteria also require an inadequate response, intolerance, or contraindication to 3 or more prescribed preventive migraine medications prior to receiving onabotulinumtoxinA treatment for the first time and appropriate management for medication-overuse headache. Continued eligibility for PBS-subsidized onabotulinumtoxinA treatment requires a maintained reduction in headache days per month of at least 50% after 2 treatment cycles (12 weeks per cycle). Patients were included in the study if they first initiated PBS-subsidized onabotulinumtoxinA treatment for prevention of migraine after 1 March 2014 and were naïve to botulinum toxin, and received at least 2 onabotulinumtoxinA treatment cycles for migraine prevention with at least 1 follow-up visit after the second cycle.

Patients receiving onabotulinumtoxinA not funded by the PBS or not for the preventive treatment of headache were excluded, as were those with headaches due to conditions other than CM or that could be attributed to another disorder. Use of any botulinum toxin other than onabotulinumtoxinA or agents that could interfere with neuromuscular transmission (e.g., aminoglycosides) was prohibited.

### Interventions

The expected treatment for each cycle was 155 U of onabotulinumtoxinA administered at 31 fixed sites as 0.1 mL (5 U) intramuscular injections and, if necessary, up to 40 U in 8 additional sites using a “follow the pain” procedure, both of which are consistent with the Therapeutic Goods Administration–approved product information [15].

Baseline (pretreatment) and follow-up (after 2 or more treatment cycles, up to 56 weeks postbaseline) data were collected from medical charts and headache diaries of eligible patients, including demographics, frequency and severity of headache and migraine days, prior and concomitant preventive and acute migraine treatments, Headache Impact Test (HIT-6) scores, and number of work or study days missed due to migraine. The HIT-6 measures the impact of headaches on daily activities with scores that range from 36 to 78, with scores of 60 and above representing severe impact [16].

### Outcomes

The primary effectiveness measure was the proportion of patients with a 50% or greater reduction in monthly headache days from baseline (i.e., PBS responders) after 2 onabotulinumtoxinA treatment cycles (i.e., approximately 6 months from baseline). Secondary endpoints included mean changes from baseline in number and severity of headache days, number of migraine days, oral preventive and acute treatment use, HIT-6 scores, missed work or study days, and medication use. A headache day was defined as a 24-h time period in which a headache lasting at least 4 continuous hours was documented in the patient diary or by the physician. Ratings of headache severity (mild, moderate, or severe) were extracted from patient diaries. No systematic prospective recording of adverse events (AEs) was undertaken. When available, information about the incidence, type, duration, and severity of treatment-related AEs and serious AEs as documented in patient records was extracted.

### Statistical methods

An estimated sample size of 150 patients was required to detect a 20% difference in the rate of response in real-world practice versus the phase 3 PREEMPT study results with power of 94% at *p* = 0.05. A target sample of 200–250 was specified to account for missing data and ensure adequately powered study evaluations. To minimize potential bias, records were screened sequentially beginning with the first patient receiving onabotulinumtoxinA treatment on or after 1 March 2014 at each study site, for a maximum of 50 patients per site.

Primary treatment effectiveness was assessed using multivariate regression analysis to adjust for potential confounders, such as age, gender, and baseline headache severity. Changes from baseline in HIT-6 score and frequencies of headache, migraine, severe headache, and missed work/study days were analysed as mean changes from baseline using a 1-tailed *t*-test (*p* ≤ 0.05) with no additional corrections made for multiple testing. Changes from baseline in the percentages of patients using various acute pain medications were analysed with the Pearson’s chi-square test (*p* < 0.05). Proportional results were reported with 95% confidence intervals, and the results for continuous variables were reported as means and standard deviations. To test the hypothesis that onabotulinumtoxinA treatment of CM was at least as effective in Australian clinics as in phase 3 PREEMPT studies, we compared the proportion of patients achieving a reduction in monthly headache days of at least 50% from baseline after 2 treatment cycles. Methods and pooled results of the 2 PREEMPT clinical trials have been previous reported [17]. The original pooled report evaluated the proportion of patients with at least a 50% reduction from baseline at the 28-day period ending with week 24. In this post hoc pooled analysis, we defined a responder as a patient achieving at least a 50% reduction from baseline in headache days in the 28-day period with the lowest headache days up to week 24 of treatment with onabotulinumtoxinA.

## Results

### Patients

Of the 236 medical records reviewed between April 2016 and January 2017, 25 records were excluded for the following reasons: the patient was not covered by the PBS (*n* = 17), the patient did not receive a second treatment cycle or was not assessed after the second cycle (*n* = 4), the assessment of headache days per month was not conducted at baseline and after 2 treatment cycles (*n* = 3), or the patient did not have CM (*n* = 1).

The mean (SD) age of the 211 patients included in the analysis was 44.6 (12.5) years at baseline, and 88.6% were women (Table 1). The mean (SD) duration of CM, which was available for 124 patients, was 14.5 (12.3) years. The mean (SD) number of headache days per month at baseline was 25.2 (5.3). The negative effect of these headaches is reflected in the mean (SD) HIT-6 score of 68.2 (4.8) from 100 patients, which is above the threshold score of 60 considered indicative of severe impact. The most commonly noted comorbidities were depression (11%), anxiety (8%), and gastroesophageal reflux disease (5%).

**Table 1:** Baseline demographics, clinical characteristics, and treatments of PBS patients

Characteristic	PBS patients *n* = 211
Age, years, mean (SD)	44.6 (12.5)
Duration of CM, mean (SD) years	14.5 (12.3)^a^
Women, n (%)	187 (88.6)
Baseline headache days/month, mean (SD)	25.2 (5.3)
Baseline migraine days/month, mean (SD)	15.3 (7.9)^b^
Baseline HIT-6 score, mean (SD)	68.2 (4.8)^c^
Patients with HIT-6 score indicating severe impact (≥60)	98.0%^d^
Previously used preventive headache medication	
Amitriptyline	152 (72.0)
Topiramate	151 (71.6)
Propranolol	143 (67.8)
Pizotifen	122 (57.8)
Sodium valproate	63 (29.9)
Verapamil	43 (20.4)
Candesartan	25 (11.8)
Cyproheptadine	19 (9.0)
Methysergide	14 (6.6)
Other: Pregabalin	12 (5.7)
Continued use of oral preventive headache medication	66.4%
Acute medication overuse at baseline^e^	61.1%
Concomitant use of simple analgesics	74.4%
Concomitant use of triptans	64.5%
Concomitant use of prescribed opioids	23.2%

CM, chronic migraine; HIT-6, 6-item Headache Impact Test; PBS, Pharmaceutical Benefits Scheme

^a^n = 124

^b^n = 197

^c^n = 100

^d^n = 100

^e^As suggested by patient’s records noting a management plan for medication overuse

The baseline patient characteristics from our sequentially screened patient records were similar to those from the population of patients who satisfied inclusion/exclusion criteria and participated in PREEMPT, with the exception of a higher mean (SD) headache day frequency and higher use of ongoing preventive headache treatments (95% CI) in the current study (25.2 [5.3] vs 19.9 [3.7] and 66.4% [60.0, 72.7] vs 0%, respectively). The median dose of onabotulinumtoxinA in both the first and most recent treatment cycle in this study was 155 U (ranges, 50–200 and 50–205, respectively). As this was a retrospective chart analysis, the number of treatment cycles and duration of follow-up periods varied. There was a median of 4 treatment cycles (range, 2–11; mean, 4.2), and the median follow-up times at the 6-month and final evaluations were 26 weeks (range, 12–83) and 60 weeks (range, 23–251), respectively.

### Headache days and impact

**Fig. 1:**
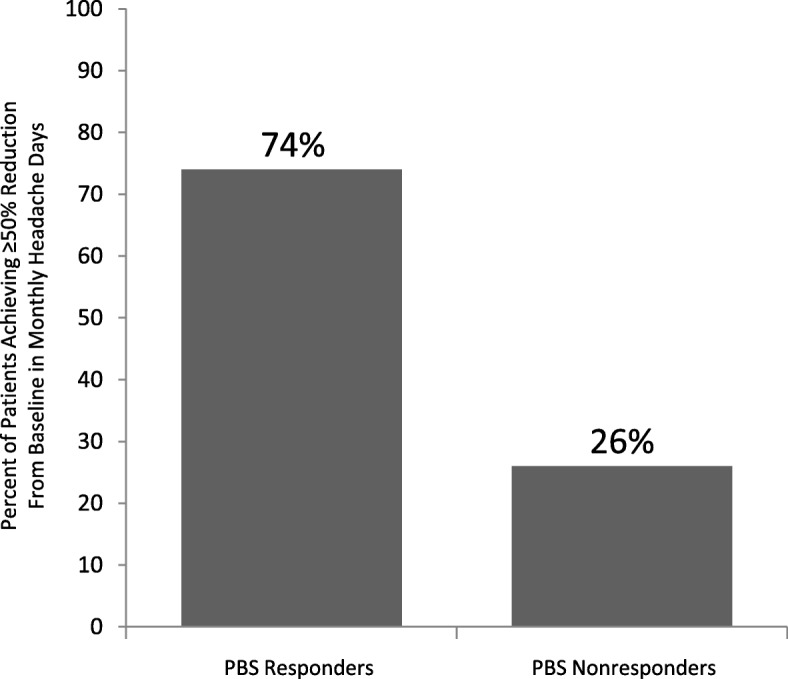
Proportion of patients achieving ≥50% reduction from baseline in monthly headache days after 2 treatment cycles. PBS, Pharmacy Benefits Scheme

**Fig. 2:**
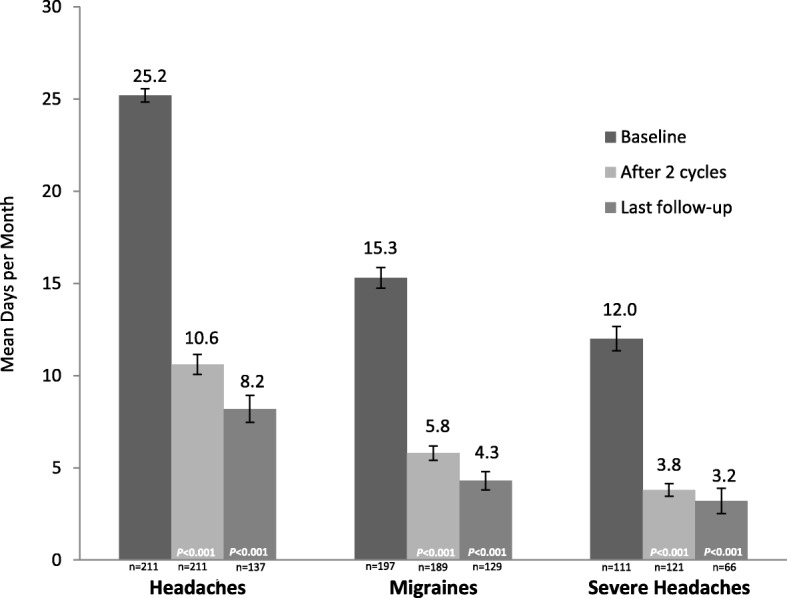
Frequency of headache, migraine, and severe headache days. The n’s represent patients with available data at each time point. *P*-values indicate significant decrease from baseline, one-sided *t*-test

In the primary outcome analysis, 74% (95% CI, 68, 80) of patients achieved a 50% or greater reduction from baseline in headache days per month after 2 treatment cycles (Fig. 1). The frequencies of headaches, migraines, and severe headaches at baseline, after 2 treatment cycles, and at last follow-up are presented in Fig. 2. The mean (SD) changes in headache days per month were − 14.7 (8.4) after 2 treatment cycles (*n* = 211; *p* < 0.001) and − 16.9 (9.0) at final follow-up (*n* = 137; *p* < 0.001). For migraine days the mean (SD) per-month reductions were − 9.4 (7.6) after 2 treatment cycles (*n* = 189; *p* < 0.001) and − 10.0 (8.4) at final follow-up (*n* = 129; *p* < 0.001). The changes in mean (SD) severe headache days per month were − 8.2 (6.8) after 2 treatment cycles (*n* = 105; *p* < 0.001) and − 8.5 (8.4) at final follow-up (*n* = 59; *p* < 0.001).

**Fig. 3:**
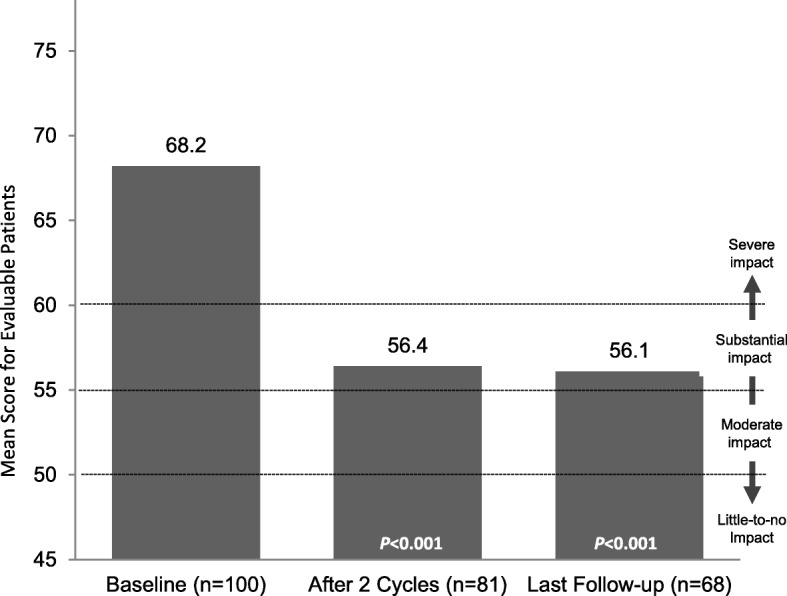
HIT-6 scores. HIT-6, 6-item Headache Impact Test. *P*-values indicate significant decrease from baseline, one-sided *t*-test

**Fig. 4:**
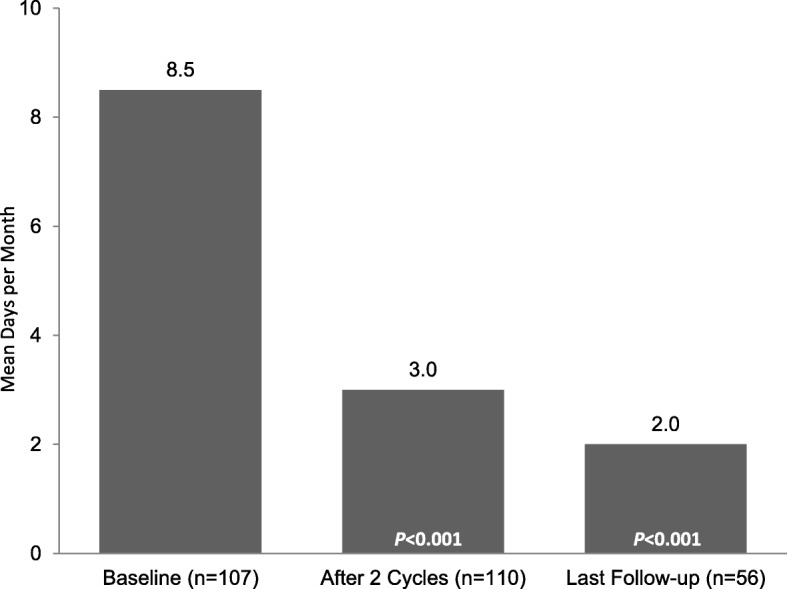
Work or study days missed due to migraine. *P*-values indicate significant decrease from baseline, one-sided *t*-test

Reductions in the adverse impact of headaches, reflected in significant mean (SD) changes in HIT-6 scores of − 11.7 (9.8) after 2 treatment cycles (*n* = 80; *p* < 0.001) and − 11.8 (12.2) at final follow-up (*n* = 68; *p* < 0.001), respectively (Fig. 3), represent a clinically meaningful reduction in HIT-6 scores. Additionally, patients reported significant mean (SD) changes from baseline in missed work or study days of − 5.0 (7.8) after 2 treatment cycles (*n* = 99; *p* < 0.001) and − 5.6 (7.7) at final follow-up (*n* = 50; *p* < 0.001) (Fig. 4).

In the post hoc analysis of the pooled PREEMPT data, 71.2% of PREEMPT patients randomized to treatment with onabotulinumtoxinA achieved a 50% or greater reduction from baseline in headache days at any point during the course of 2 treatment cycles. Comparatively, only 62.6% of PREEMPT patients randomized to placebo exhibited a 50% or greater reduction from baseline in headache days when the same analysis was applied to this group.

### Concomitant medications

**Fig. 5:**
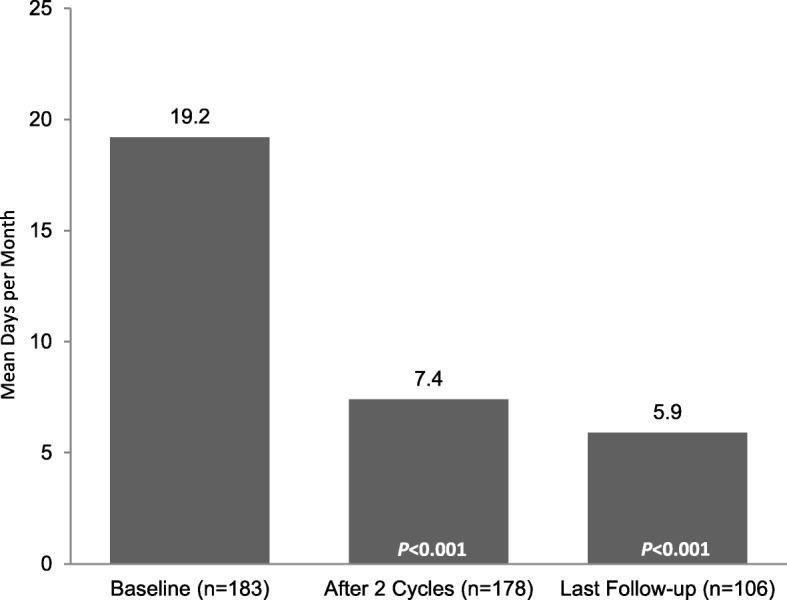
Days of acute pain medication use. *P*-values indicate significant decrease from baseline, one-sided *t*-test

**Fig. 6:**
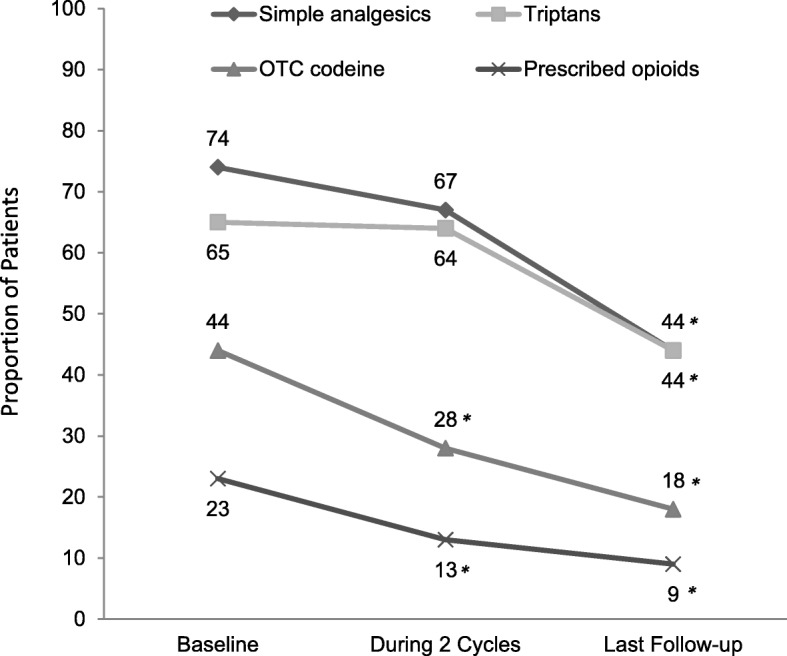
Acute pain medication usage by type. OTC, over the counter. *P < 0.050, Pearson’s Chi-squared test

The proportion of patients using oral preventive medications decreased from 66% (95% CI: 60, 73) at baseline to 51% (95% CI: 44, 57) at final follow-up. The oral preventive medications used most commonly prior to this study were amitriptyline (72%), topiramate (72%), and propranolol (68%). There were significant mean (SD) declines in acute pain medication use of − 11.5 (7.6) days per month after 2 treatment cycles (*n* = 167; *p* < 0.001) and − 12.7 (8.1) days at final follow-up (*n* = 103; *p* < 0.001) (Fig. 5). Reduced use of acute pain medications also was evident by statistically significant declines in the proportions of patients using simple analgesics, triptans, and opioids (Fig. 6); the largest decreases in use were noted in patients taking prescription or OTC formulations of opioids at baseline, with fewer than half of these patients continuing to report opioid use at last follow-up.

### Safety

In this retrospective study, no systematic prospective recording of adverse events was undertaken. Review of the medical records revealed no serious adverse events attributable to onabotulinumtoxinA.

## Discussion

In this retrospective review of medical records from 211 patients with CM treated with PBS-subsidized onabotulinumtoxinA in Australia, the data showed a reduction in headache frequency at 2 treatment cycles and beyond. Patients also experienced reductions in monthly severe headache days, monthly migraine days, HIT-6 scores, missed days of work or study, and use of acute pain medications, including opioids. These results demonstrate the clinical benefit of onabotulinumtoxinA treatment to the lives of Australian patients with CM under real-world conditions.

The phase 3 PREEMPT trials demonstrated that onabotulinumtoxinA significantly reduces the frequency of headache and migraine days compared with placebo [17]. Baseline demographic differences between the populations in this study and the PREEMPT trials exist due to the stricter inclusion and exclusion criteria of the phase 3 PREEMPT registration trials versus the less-restrictive requirements for PBS-subsidized onabotulinumtoxinA treatment. Regardless, both this study and the PREEMPT trials included patients with a broad range of ages (19–81 years and 18–65 years, respectively) and a median duration of CM longer than a decade. The observed effectiveness reported in this study was found in a patient population with a median duration of CM of 19.2 years, compared with 14.5 years in PREEMPT. Furthermore, PREEMPT inclusion criteria required 4 distinct headache attacks lasting at least 4 h, which excluded persons with continuous headache during the 4-week baseline period and excluded those who used any oral preventive treatment within 28 days of the start of baseline.

The present study had a higher proportion of patients with concomitant use of prescribed opioids. During the time the individuals in the present study were treated, codeine up to 15 mg per tablet in combination with simple analgesics was available without a prescription. Codeine-based medication-overuse headache has been a relatively common problem in Australia [18, 19]. The reductions in acute pain medication use seen in this analysis were statistically significant and may be clinically important, given the existing societal issues surrounding opioid dependence.

These findings are in agreement with previously published efficacy results from the phase 3 PREEMPT trials [17, 20, 21] and several observational studies [22–25] that have documented the efficacy and tolerability of onabotulinumtoxinA for preventive treatment of CM across Europe and North America. In the present study’s population of patients who had not experienced satisfactory results in the course of at least 3 previous treatments, the proportion achieving 50% or greater reduction in headache days per month was significantly greater than the 50% response rate after 2 treatment cycles in the phase 3 PREEMPT trials (74% vs 47%, respectively; *p* < 0.001) [17]. This difference may be, at least in part, due to differences in analysis methodology between real-world practice and clinical trials. Within the PREEMPT trials the 50% endpoint assessment was strictly limited to the 28-day period ending with week 24 (2 treatment cycles) [11, 12, 17], in contrast to the broader time period allowed for in clinical practice, which has no set time points for headache day reduction assessments. In the post hoc analysis of pooled PREEMPT trial data with an expanded response analysis period, which is consistent with the PBS criteria, 71% of PREEMPT patients receiving onabotulinumtoxinA experienced a 50% or greater decrease in monthly headache days by week 24 (2 treatment cycles). This analysis more closely matches the results seen in the current real-world study (74%).

The COMPEL study was an international, multicenter, phase 4, open-label, long-term prospective study to evaluate the long-term efficacy of onabotulinumtoxinA in adults with CM that included treatment sites in the United States, Australia, and South Korea [13]. Although primary research goals differed significantly from the COMPEL study, the present retrospective study included patients with higher mean baseline headache days (22.0 vs 25.2) and showed an overall greater mean reduction in monthly headache days at 24 weeks (− 7.4 vs − 14.7) [13].

In a post-hoc analysis of PREEMPT it was shown that 49.3% of patients had a 50% or greater reduction in headache days during the first treatment cycle [21]. After the second treatment cycle, 11.3% of patients reported a first-time response, and an additional 10.3% of patients reported a first-time response following the third treatment cycle. Therefore, the cumulative percentages of PREEMPT patients who achieved a response following the second and third treatment cycles were 60.6% and 70.9%, respectively. These cumulative responder rates are more in line with the results of the present study.

A contributing factor to the improved response rate at 2 treatment cycles in private practice in Australia in comparison to the PREEMPT trials might include the Australian practitioners having an increased familiarity with injection techniques since the PREEMPT trials were conducted. Concomitant use of preventive headache medication also may have contributed to improved response in the present study. Although inadequate response, intolerance, or contraindication to at least 3 preventive medications was required for study inclusion, those experiencing an inadequate response may have continued taking a preventive treatment to maintain a perceived partial improvement; however, patients in the COMPEL study receiving stable preventive treatment at baseline had a significantly smaller reduction in headache days compared with those not receiving preventive medications [13].

The primary limitations of this study are its retrospective, pre-post study design and the lack of a control group. Variability across treatment sites with respect to patient selection, data collection, and treatment practices is likely to be higher for retrospective studies than prospective trials. Less-stringent exclusion criteria in the present study compared with clinical trials likely produced a more diverse treatment population, and the individualized practices among clinicians for patient selection and prescribing practices may have increased or moderated this effect. Data availability varied between study centres, such that the sample sizes of various outcome measures differed across time. Finally, the reporting of AEs in this study was based on AEs documented in charts during the evaluation period, which differs from the standardized AE-assessment practices in randomized clinical trials.

The real-world design of this study is a strength because it assesses the effectiveness (i.e., efficacy and tolerability) and not just the efficacy of onabotulinumtoxinA treatment in patients with CM in the typical clinical setting. Additional strengths are the large, diverse sample and its high degrees of disease burden and unmet treatment need at baseline, as reflected by a median of 28 headache days per month, a mean HIT-6 score reflective of severe impact, and the inability to achieve adequate improvement with at least 3 prior medications. Finally, the concordance of these effectiveness findings with results from other onabotulinumtoxinA clinical and real-world studies [17, 20–25] reinforces the validity of this study and adds to the pool of evidence supporting onabotulinumtoxinA treatment for CM.

## Conclusions

In this population of Australians with CM who did not achieve adequate results with at least 3 previous preventive migraine medications, onabotulinumtoxinA treatment produced reductions in migraine days, severe headache days, HIT-6 scores, and missed work or study, compared to baseline. Nearly three-quarters of patients had their number of monthly headache days cut at least in half in the first 6 months. The benefits of treatment with onabotulinumtoxinA were sustained up to the patients’ final follow-up visit, with a trend toward further improvement after the first 2 treatment cycles. This retrospective analysis of patients with CM adds to the worldwide body of clinical evidence supporting the utility of onabotulinumtoxinA for CM prevention. Furthermore, the real-world study approach shows that onabotulinumtoxinA continues to show effectiveness in the Australian patient with migraine for whom previous preventive treatments proved inadequate.

## Data Availability

Data reported in this manuscript are available within the article. Additional data from the study may be requested at http://www.allerganclinicaltrials.com/PatientDataRequest.htm.

## Abbreviations

AE: Adverse events
CM: Chronic migraine
COMPEL: Chronic Migraine OnabotulinumtoxinA Prolonged Efficacy Open-Label
HIT-6: Six-item Headache Impact Test
PBS: Pharmaceutical Benefits Scheme
PREEMPT: Phase 3 Research Evaluating Migraine Prophylaxis Therapy
